# Impact of an exercise program combined with dietary advice on avoiding insulin prescription in women with gestational diabetes: a randomized controlled trial

**DOI:** 10.1186/s13098-024-01470-1

**Published:** 2024-09-30

**Authors:** Michel Boulvain, Véronique Othenin-Girard, François R. Jornayvaz, Bengt Kayser

**Affiliations:** 1 Faculty of Medicine, University of Geneva, Geneva, Switzerland; 2 Department of Gynaecology and Obstetrics, Geneva University Hospitals, Geneva, Switzerland; 3 Division of Endocrinology, Diabetes, Nutrition and Patient Therapeutic Education, Geneva University Hospitals, Geneva, Switzerland; 4 Institute of Sport Sciences, University of Lausanne, Synathlon-Uni-Centre, Lausanne, 1015 Switzerland

**Keywords:** Exercise, Gestational diabetes, Randomised trial, Insulin

## Abstract

**Objective:**

To assess the effectiveness of an exercise intervention, in addition to standard care, in preventing or delaying insulin prescription in women with gestational diabetes mellitus (GDM).

**Design:**

Randomised controlled trial.

**Setting:**

University hospital.

**Population:**

Pregnant women at 25–35 weeks of gestation diagnosed with GDM.

**Methods:**

Women in the intervention group participated in weekly, supervised, 30–45 min exercise sessions and were encouraged to accumulate more than 5000 steps per day, tracked by a pedometer, in addition to receiving usual care. The control group received standard care only.

**Main outcome measure:**

Insulin prescription.

**Results:**

From February 2008 through April 2013, 109 women were randomized into the intervention group (*n* = 57) or the usual care group (*n* = 52). Two women in the intervention group were excluded from the analysis (one was randomised in error and one was lost to follow-up). Six women never attended the exercise sessions, and two attended fewer than two sessions. However, two-third of women were considered as compliant to the intervention (attended more than 50% of sessions and/or averaged more than 5000 steps/day). The incidence of insulin prescription did not differ between the groups: 31 women (56%) in the intervention group versus 24 women (46%) in the control group (RR 1.22, 95% CI 0.84 to 1.78). The median time from randomization to insulin prescription was also similar between groups (14 days in the intervention group and 13 days in the control group).

**Conclusion:**

This study did not demonstrate that an exercise program reduces or delays insulin prescription in women with GDM. Low adherence to the intervention, a small sample size, and the short duration of the program may explain the lack of observed benefit.

**Registered:**

At clinicaltrials.gov, NCT03174340, 02/06/2017

## Introduction

Gestational diabetes mellitus (GDM) is defined as diabetes diagnosed during the second or third trimester of pregnancy that was not clearly present prior to gestation [1]. Globally, GDM affects approximately 14% of pregnancies, though prevalence varies depending on the population and the diagnostic criteria used. In Geneva, Switzerland, 11% of pregnant women are diagnosed with GDM according to the IADPSG criteria [21].

The initial management of GDM typically involves dietary guidance, physical activity advice, and regular glucose monitoring. If euglycemia cannot be achieved by lifestyle interventions alone, insulin therapy is prescribed [12].

Exercise has been well-documented to improve insulin sensitivity in non-diabetic individuals [20], with the most pronounced benefits observed in milder forms of type 2 diabetes [25], a condition closely related to GDM.

A systematic review of observational studies found that women who engage in regular physical activity before or during pregnancy have a lower risk of developing GDM [24]. Additionally, randomized trials evaluating early pregnancy exercise interventions have demonstrated that physical activity can help prevent the onset of GDM [15, 23].

In women already diagnosed with GDM, increasing daily moderate physical activity may improve glycaemic control and decrease the need for insulin therapy [13]. For many women, the prospect of insulin therapy is a source of stress and anxiety [11], as it requires regular injections, glucose monitoring and dose adjustments, which are often perceived as an added burden [9]. Moreover, increasing physical activity in women with GDM has been linked to improved maternal health, a reduced risk of caesarean section, and decreased maternal and neonatal morbidity associated with GDM [8].

Previous research on exercise interventions for women with GDM included 11 trials with 638 participants, summarized in a systematic review [4]. These trials reported reductions in fasting and post-prandial glycaemia following exercise interventions, although there was little evidence of improvement in clinically significant outcomes. One study that incorporated both supervised and home-based exercise sessions suggested a reduction in insulin prescriptions [6], while others primarily demonstrated improvements in glycaemia control [4]. Recent reviews concluded that exercise may be beneficial for managing GDM, but more research is needed to confirm these effects [7].

Given the need for further evidence, we designed a randomized clinical trial (RCT) to assess the impact of an exercise intervention on insulin prescription rates in women with GDM in our university hospital outpatient clinic. We selected insulin prescription as the primary outcome, as it reflects the threshold at which lifestyle interventions fail to control glycaemia. Since many women in our clinic face time and travel constraints that make intensive exercise schedules difficult, we designed a flexible, low-cost, once-weekly exercise program that could be incorporated into their routine. Our objective was to determine whether this program could reduce the need for insulin therapy in women with GDM.

## Methods

### Study design and participants

We conducted a randomized controlled trial in the Geneva University Hospitals (Switzerland) from February 2008 through April 2013. Women recently diagnosed with gestational diabetes using standard criteria [5] and referred to a multidisciplinary team of diabetologists, specialised nurses, dieticians, obstetricians and midwives were invited to participate. From 2008 until 2010, GDM was diagnosed with a 50 g OGTT (O’Sullivan test: [19], where a 50 g OGTT ≥ 11 mmol/L was considered as gestational diabetes. If the result was between 7.8 mmol/L and 11 mmol/L, a 100 g OGTT was performed, with results interpreted using the Carpenter and Coustan criteria [5]. From 2011 onward, GDM was diagnosed with a 75 g OGTT with IADPSG criteria [14] following the recommendations from the Swiss Society of Obstetricians and Gynecologists. Consenting women with a singleton pregnancy, a positive GDM test, and no insulin or oral antidiabetic treatment were eligible to participate. Exclusion criteria included age below 18 years, insulin treatment prescribed before or at the first visit, pre-existing diabetes, and any contraindication for physical activity [2].

### Randomisation and masking

Women were randomized to the intervention or usual care group using a list of randomly permuted blocks (block size of four to eight), distributed in opaque, consecutively numbered, sealed envelopes. Clinicians and participants had no access to the randomisation list but were not blinded to group allocation, which was disclosed after inclusion. The diabetologists responsible for insulin prescription during follow-up were, to the extent possible, blinded to group assignment.

### Procedures

The research team approached pregnant women during their first prenatal consultation after the GDM diagnosis. These women either followed up their pregnancy at the hospital or were referred by their private practitioner. Eligible women were informed of the trial during this visit, in addition to receiving standard GDM management information. After consenting, participants were randomly allocated to one of the two groups. Women in both groups received usual care, which included dietary advice, exercise recommendations, and capillary blood glucose self-monitoring four times daily [10]. In the intervention group, an exercise program was added to usual care. This program involved weekly, supervised, in-hospital exercise sessions, coinciding with their clinic appointments for GDM management. Each session lasted 30–45 min and included a combination of endurance exercise (stationary cycling, arm-cranking) and light resistance training (elastic bands and free weights), tailored to individual preferences and tolerance. The sessions started with a light load arm-cranking, followed by 10 min of arm-cranking targeting a heart rate > 130 bpm, which was chosen as it reflects the lower boundary of recommended intensity for unfit women [17]. After a 5-minute rest, participants engaged in 20 min of recumbent cycling, also targeting a heart rate > 130 bpm. If cycling was uncomfortable, stepping exercise was used as a substitute. A heart rate monitor (Suunto Smartbelt, Vantaa, Finland) was worn during sessions, and intensity was measured by the average heart rate.

The physical therapist used motivational interviewing techniques [22] to encourage participants to increase daily physical activity. The physical therapist, trained by a motivational interviewing technique instructor, emphasized open-ended questions, affirmations, reflections, and summaries to facilitate change. Participants were encouraged to accumulate 5000 or more steps daily, monitored by a pedometer (HJ 112, Omron, Hoofddorp, Netherlands), as 5000 steps per day is the threshold for a sedentary lifestyle [26]. Participants recorded their physical activities, daily step counts, and blood glucose levels in a diary. The intervention continued until the end of the pregnancy.

### Outcomes

Compliance with the exercise program was defined as attending at least 50% of scheduled weekly exercise sessions between randomization and delivery and/or averaging more than 5000 steps per day. The primary outcome was the incidence of insulin prescription. Women who did not achieve glycaemic targets (≤ 5.3 mmol/l fasting, ≤ 8.0 mmol/l one hour postprandially) were treated with basal and/or prandial insulin, in according with the Swiss Society for Endocrinology and Diabetes, adapted from the American Diabetes Association recommendations [1]. Insulin treatment included intermediate-acting NPH insulin, typically initiated at bedtime (0.1 U/kg/day), and short-acting insulin (aspart or lispro) at mealtime. Oral antidiabetic agents were not used. Secondary outcomes included time to insulin, maximum dose of insulin, mode of delivery, birthweight and neonatal morbidity.

### Statistical analysis

Analyses were conduction on an intention-to-treat basis. We report baseline characteristics and outcomes as means (SD), medians (IQR), or percentages. The effects of the intervention were assessed using relative risks (RR) with 95% confidence intervals (CI). Statistical significance was determined using Fisher’s exact test for categorical variables and Student’s T-test for continuous variables. We used SPSS (versions 18 and 20, IBM, Chicago, USA) and Stata (version 15, StataCorp, College Station, USA) for statistical analysis. Based on clinic data, we anticipated an 40% incidence of insulin prescription in the control group. To detect a clinically relevant reduction to 20% in the intervention group (number-needed-to-treat of 5), we calculated a required sample size of 91 patients per group (α = 0.05, 80% power). However, the trial was stopped prematurely due to low recruitment, poor compliance with the exercise sessions, and a lack of funding.

## Results

**Fig. 1:**
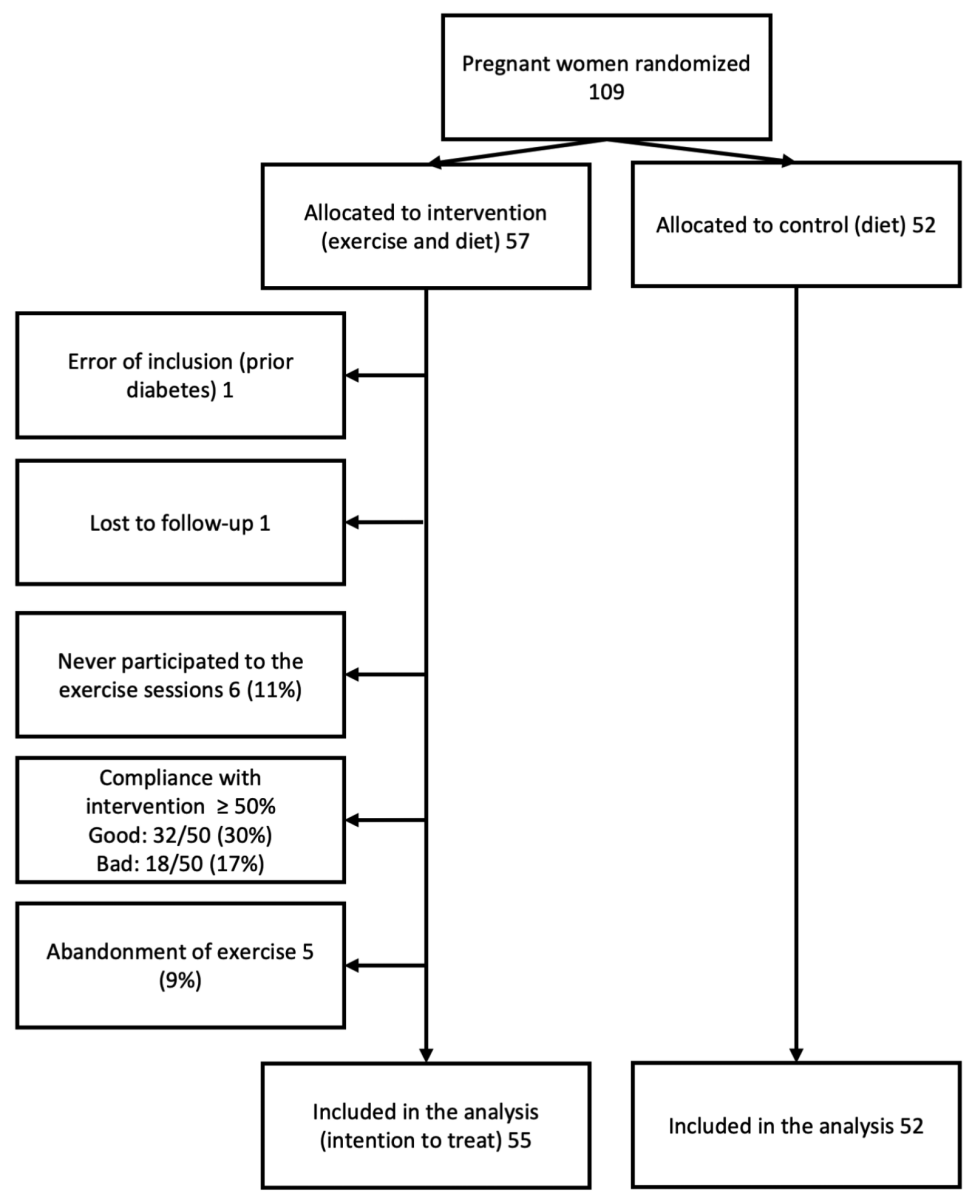
Flow diagram

From February 2008 through April 2013, 109 consenting women were randomized, with 57 allocated to the exercise group and 52 to the usual care group (Fig. 1). One woman was excluded because of diabetes type 1, and another was lost to follow-up (delivered at another hospital), leaving 107 women for analysis.

Baseline characteristics are summarised in Table 1. There were more obese women (BMI 30 kg/m^2^ or more) in the intervention group, and more nulliparous women in the usual care group.

**Table 1:** Maternal characteristics at randomization

	Exercise	Control
	*n* = 55	*n* = 52
Maternal age (years): mean (SD)	33.0 (5.3)	33.8 (6.5)
Nulliparous women: n (%)	28 (50.9%)	35 (67.3%)
Smoker: n (%)	8/53 (15.1%)	7/49 (14.3%)
Ethnic group: n (%)		
Caucasian	43 (78.2%)	36 (69.2%)
Black	11 (20.0%)	10 (19.2%)
Other	1 (1.8%)	6 (11.5%)
Weight before pregnancy (kg): mean (SD)	68.9 (18.3)	65.8 (12.6)
Weight at randomisation (kg): mean (SD)	79.7 (16.4)	75.6 (11.7)
Weight gain at randomisation (kg): mean (SD)	10.8 (5.3)	9.8 (4.2)
Height (cm): mean (SD)	162.3 (5.8)	161.8 (7.0)
BMI (kg/m^2^): mean (SD)	26.1 (6.5)	25.1 (4.4)
BMI categories: n (%)		
Normal (< 25 kg/m^2^)	32 (58.2%)	27 (51.9%)
Overweight (25 to < 30 kg/m^2^)	10 (18.2%)	20 (38.5%)
Obese (30 to < 35 kg/m^2^)	8 (14.6%)	4 (7.7%)
Morbidly obese (35 or more kg/m^2^)	5 (9.1%)	1 (1.9%)
Gestational age at randomization (weeks): mean (SD)	29.7 (2.1)	29.8 (2.4)
Type of screening: n (%)		
O’Sullivan, followed by OGTT 100 gr	26 (47.3%)	23 (44.2%)
OGTT 75 gr	29 (52.7%)	29 (55.8%)

In the intervention group, six women (11%) never attended the exercise sessions and two women (4%) participated minimally (one session). Thirty-five women (64%) were considered compliant with the intervention: 32 attended more than 50% of sessions, and three did not but averaged more than 5000 steps per day. The average session duration was 43 (SD 5) minutes, with an average heart rate of 114 (SD 12) bpm and a peak heart rate of 142 (SD 17) bpm. No adverse events occurred during the exercise sessions.

The incidence of insulin prescription did not differ between groups: 31 women (56%) in the exercise group versus 24 (46%) in the control group (RR 1.22, 95% CI 0.84 to 1.78; *P* = 0.39) (Table 2).

The time between randomization and insulin initiation was also similar: 16 (SD 13) days in the exercise group versus 17 (SD 15) days in the usual care group (*P* = 0.62). Maximal insulin doses were comparable between the two groups. Between 26 and 32 weeks, mean postprandial glucose was 9.4 (SD 1.8) mmol/L in the exercise group compared to 8.9 (SD 2.0) mmol/L in the usual care group (*P* = 0.22). The number of postprandial glucose readings above 8.0 mmol/L was similar between groups (4.1, SD 3.4 and 3.8, SD 3.1; *P* = 0.77). From 32 to 36 weeks, mean postprandial glucose was 9.0 (SD 2.1) mmol/L in the exercise group compared to 8.9 (SD 1.8) mmol/L in the usual care group, (*P* = 0.75), and the number of postprandial glucose readings above 8.0 mmol/L remained similar (3.9, SD 3.4 and 4.1, SD 3.4; *P* = 0.84).

Secondary outcomes, including gestational weight gain, caesarean section rate, maternal complications, did not differ significantly between groups. Neonatal outcomes were also similar (Table 2).

**Table 2:** Maternal and neonatal outcomes. Results are presented as n (%), except when stated otherwise

	Exercise	Control	RR (95%CI) or *P*-value
	*n* = 55	*n* = 52	
Insulin prescription	31 (56.4%)	24 (46.2%)	1.22 (0.84–1.78)
Delay before insulin prescription (days): mean (SD)	16 (13)	17 (15)	0.62
Maximum dose of insulin (Units/day): mean (SD)			
Short acting	7.3 (9.6)	5.0 (4.8)	0.26
Intermediate acting	9.4 (11.2)	8.5 (4.4)	0.67
Weight gain from randomization to delivery (kg): mean (SD)	3.4 (4.1)	3.5 (2.8)	0.93
Preterm delivery	5 (9.1%)	5 (9.6%)	0.95 (0.29–3.08)
Induction of labour	31 (56.4%)	35 (67.3%)	0.84 (0.62–1.13)
Caesarean section	22 (40.0%)	13 (25.0%)	1.60 (0.90–2.83)
Vaginal delivery	33 (60.0%)	39 (75.0%)	
Spontaneous	25	31	
Instrumental	8	8	
Birthweight (grams): mean (SD)	3337 (559)	3245 (459)	0.35
Macrosomia (4000 gr or more)	11 (20.0%)	6 (11.5%)	0.29
Apgar score less than 7 at 5’	1	0	
Neonatal resuscitation	2	2	
Neonatal complications	5	6	
Jaundice	1	2	
Respiratory distress	2	2	
Others	2	2	

## Discussion

We aimed to test whether a voluntary, lightweight, once-weekly supervised exercise intervention, combined with advice to increase physical activity at home could prevent or delay the need for insulin in women diagnosed with gestational diabetes (GDM). Unfortunately, our findings did not demonstrate that this program improved glycaemic control sufficiently to prevent or delay insulin prescription. Additionally, none of the secondary outcomes, such as time to insulin initiation, maximum insulin dose, mode of delivery, birthweight, or neonatal morbidity, were significantly affected by the intervention.

One possible explanation for the lack of efficacy is poor compliance. Due to travel and time constraints, women attending our university clinic struggled to participate in even one weekly supervised exercise session, which led to low overall adherence in the intervention group. Moreover, an imbalance in baseline characteristics between the groups–likely due to chance–could have influenced the results. The fact that the diabetologists responsible for insulin prescriptions were not fully blinded to the group assignments may have also affected the outcomes.

Our negative results are in line with other studies and recent reviews and meta-analyses that similarly found no significant impact of an exercise intervention on the prevention of insulin use in women with GDM [23].

Exercise before, during, and after pregnancy provides numerous health benefits for mothers, including improved cardiovascular function and prevention of conditions like preeclampsia, GDM, varicose veins, deep vein thrombosis, lower back pain, and enhanced psychological wellbeing [18]. It also limits weight gain during pregnancy and reduces post-partum fat retention [3]. Furthermore, regular physical activity decreases the risk of preterm delivery, shortens labour duration, and reduces perinatal complications [18]. Offspring of physically active mothers tend to have lower birth weight, reduced foetal body fat, and improved early childhood health [16]. Women who are physically active prior to pregnancy have a lower risk of GDM, and starting regular exercise early in pregnancy can help prevent its onset [16].

While some studies that implemented 3 exercise sessions per week reported clinically relevant effects [4], women in our setting face limitations to time and travel, preventing more frequent attendance. Therefore, we assessed whether a lightweight intervention, combined with a recommendation to accumulate at least 5000 steps per day, could achieve a meaningful clinical outcome. The lack of benefit in our study may be due to the insufficient frequency and intensity of exercise.

Although some centres use oral medications to manage GDM, our centre relies on insulin therapy, which is considered safe for the foetus as it does not cross the placenta. However, insulin treatment carries risks such as hypoglycaemia, is costly, and requires specialized instruction from nurses. Moreover, many women are hesitant to self-administer insulin injections [9]. Thus, if physical activity could prevent the need for insulin, this would simplify GDM management and reduce associated healthcare costs.

The limitations of our study could account for absence of significant effects. In many cases, insulin was prescribed just a few weeks after the start of the exercise program, limiting the potential for the intervention to show its full impact. We also had to halt recruitment before reaching the calculated sample size, reducing the statistical power to detect a difference. Additionally, the exercise program was relatively light, with short duration, and the cut-off for compliance of 50% was set too low. We did not monitor physical activity changes in the control group, which limits our ability to assess a potential Hawthorne effect–where participants may have improved simply due the their awareness of being observed. Future studies should include physical activity questionnaires for both groups to evaluate differences attributable solely to the intervention.

Moreover, a formal assessment of the quality assessment of the motivational interviewing techniques used by the physical therapist could enhance future interventions, as this approach was shown to be effective when properly employed [22].

## Conclusion

Our light-weight intervention, designed for large-scale applicability with minimal resources, did not significantly reduce the need for insulin prescription or improve secondary outcomes. This study was underpowered to detect the potential effects of such a modest exercise intervention. Starting regular physical activity earlier in pregnancy has shown success in preventing GDM, but whether initiating an exercise program after a GDM diagnosis is too late to make a difference remains to be determined by larger, more robust studies. We recommend future trials explore more frequent and intense exercise regimens and assess adherence to physical activity guidelines more comprehensively.

## Data Availability

The datasets used and analysed during the current study are available from Michel Boulvain (boulvain.michel@gmail.com), on reasonable request.

## References

[1] ADA. Gestational diabetes mellitus. Diabetes Care. 2004;27(Suppl 1):S88–90. 14693936 10.2337/diacare.27.2007.s88

[2] Artal R, O’Toole M. Guidelines of the American college of obstetricians and gynecologists for exercise during pregnancy and the postpartum period. Br J Sports Med. 2003;37(1):6–12. 12547738 10.1136/bjsm.37.1.6 PMC1724598

[3] Blaize AN, Pearson KJ, Newcomer SC. Impact of maternal exercise during pregnancy on offspring chronic disease susceptibility. Exerc Sport Sci Rev. 2015;43(4):198–203. 10.1249/JES.0000000000000058 PMC4575625 26196867

[4] Brown J, Ceysens G, Boulvain M. Exercise for pregnant women with gestational diabetes for improving maternal and fetal outcomes. Cochrane Database Syst Rev. 2017;6:CD012202. 28639706 10.1002/14651858.CD012202.pub2 PMC6481507

[5] Coustan DR, Carpenter MW. The diagnosis of gestational diabetes. Diabetes Care. 1998;21(Suppl 2):B5–8. 9704220

[6] de Barros MC, Lopes MA, Francisco RP, Sapienza AD, Zugaib M. Resistance exercise and glycemic control in women with gestational diabetes mellitus. Am J Obstet Gynecol. 2010;203(6):556.e1-6. 10.1016/j.ajog.2010.07.015 20864072

[7] Dingena CF, Arofikina D, Campbell MD, Holmes MJ, Scott EM, Zulyniak MA. Nutritional and exercise-focused lifestyle interventions and glycemic control in women with diabetes in pregnancy: a systematic review and meta-analysis of randomized clinical trials. Nutrients. 2023;15(2):323. 10.3390/nu15020323 PMC9864154 36678193

[8] Domenjoz I, Kayser B, Boulvain M. Effect of physical activity during pregnancy on mode of delivery. Am J Obstet Gynecol. 2014;211(4):e401401–11. 10.1016/j.ajog.2014.03.030 24631706

[9] Figueroa Gray M, Hsu C, Kiel L, Dublin S. It’s a very big burden on me: women’s experiences using insulin for gestational diabetes. Matern Child Health J. 2017;21(8):1678–85. 10.1007/s10995-017-2261-8 28092062

[10] Gariani K, Egloff M, Prati S, Philippe J, Boulvain M, Jornayvaz FR. Consequences of the adoption of the IADPSG versus Carpenter and Coustan criteria to diagnose gestational diabetes: a before-after comparison. Exp Clin Endocrinol Diabetes. 2019;127(7):473–6. 30257263 10.1055/a-0735-9469

[11] Hui AL, Sevenhuysen G, Harvey D, Salamon E. Stress and anxiety in women with gestational diabetes during dietary management. Diabetes Educ. 2014;40(5):668–77. 24874692 10.1177/0145721714535991

[12] Kjos SL, Buchanan TA. Gestational diabetes mellitus. N Engl J Med. 1999;341(23):1749–56. 10580075 10.1056/NEJM199912023412307

[13] Martinez-Vizcaino V, Sanabria-Martinez G, Fernandez-Rodriguez R, Cavero-Redondo I, Pascual-Morena C, Alvarez-Bueno C, Martinez-Hortelano JA. Exercise during pregnancy for preventing gestational diabetes mellitus and hypertensive disorders: an umbrella review of randomised controlled trials and an updated meta-analysis. BJOG. 2023;130(3):264–75. 36156844 10.1111/1471-0528.17304 PMC10092296

[14] Metzger BE, Gabbe SG, Persson B, Buchanan TA, Catalano PA, Damm P, Dyer AR, Leiva A, Hod M, Kitzmiler JL, Lowe LP, McIntyre HD, Oats JJ, Omori Y, Schmidt MI. International association of diabetes and pregnancy study groups recommendations on the diagnosis and classification of hyperglycemia in pregnancy. Diabetes Care. 2010;33(3):676–82. 20190296 10.2337/dc09-1848 PMC2827530

[15] Ming WK, Ding W, Zhang CJP, Zhong L, Long Y, Li Z, Sun C, Wu Y, Chen H, Chen H, Wang Z. The effect of exercise duringpregnancy on gestational diabetes mellitus in normal-weight women: a systematic review and meta-analysis. BMC PregnancyChildbirth. 2018;18(440). 10.1186/s12884-018-2068-7. 10.1186/s12884-018-2068-7 PMC6233372 30419848

[16] Mottola MF, Artal R. Role of exercise in reducing gestational diabetes mellitus. Clin Obstet Gynecol. 2016;59(3):620–8. 10.1097/GRF.0000000000000211 27135873

[17] Mottola MF, Davenport MH, Brun CR, Inglis SD, Charlesworth S, Sopper MM. VO2peak prediction and exercise prescription for pregnant women. Med Sci Sports Exerc. 2006;38(8):1389–95. 16888450 10.1249/01.mss.0000228940.09411.9c

[18] Muktabhant B, Lawrie TA, Lumbiganon P, Laopaiboon M. Diet or exercise, or both, for preventing excessive weight gain in pregnancy. Cochrane Database Syst Rev. 2015;(6):CD007145. 10.1002/14651858.CD007145.pub3 PMC9428894 26068707

[19] O’Sullivan JB, Mahan CM. Criteria for the oral glucose tolerance test in pregnancy. Diabetes. 1964;13:278–85. 14166677

[20] Ronnemaa T, Mattila K, Lehtonen A, Kallio V. A controlled randomized study on the effect of long-term physical exercise on the metabolic control in type 2 diabetic patients. Acta Med Scand. 1986;220(3):219–24. 3535397 10.1111/j.0954-6820.1986.tb02754.x

[21] Ryser Ruetschi J, Jornayvaz FR, Rivest R, Huhn EA, Irion O, Boulvain M. Fasting glycaemia to simplify screening for gestational diabetes. BJOG. 2016;123(13):2219–22. 10.1111/1471-0528.13857 26810795

[22] Smith R, Ridout A, Livingstone A, Wango N, Kenworthy Y, Barlett K, Coburn H, Reid H, Jones N, Mackillop L. Motivational interviewing to increase physical activity in women with gestational diabetes. Br J Midwifery. 2021;29(10):550–6.

[23] Sweeting A, Hannah W, Backman H, Catalano P, Feghali M, Herman WH, Hivert MF, Immanuel J, Meek C, Oppermann ML, Nolan CJ, Ram U, Schmidt MI, Simmons D, Chivese T, Benhalima K. Epidemiology and management of gestational diabetes. Lancet. 2024;404(10448):175–92. 10.1016/S0140-6736(24)00825-0 38909620

[24] Tobias DK, Zhang C, van Dam RM, Bowers K, Hu FB. Physical activity before and during pregnancy and risk of gestational diabetes mellitus: a meta-analysis. Diabetes Care. 2011;34(1):223–9. 20876206 10.2337/dc10-1368 PMC3005457

[25] Trovati M, Carta Q, Cavalot F, Vitali S, Banaudi C, Lucchina PG, Fiocchi F, Emanuelli G, Lenti G. Influence of physical training on blood glucose control, glucose tolerance, insulin secretion, and insulin action in non-insulin-dependent diabetic patients. Diabetes Care. 1984;7(5):416–20. 6389056 10.2337/diacare.7.5.416

[26] Tudor-Locke C, Craig CL, Thyfault JP, Spence JC. A step-defined sedentary lifestyle index: <5000 steps/day. Appl Physiol Nutr Metab. 2013;38(2):100–14. 23438219 10.1139/apnm-2012-0235

